# Gallbladder carcinosarcoma accompanied with bile duct tumor thrombi: A case report

**DOI:** 10.3892/ol.2013.1289

**Published:** 2013-04-04

**Authors:** YAN WANG, XIAODONG GU, ZHENYANG LI, JIANBIN XIANG, ZONGYOU CHEN

**Affiliations:** Department of General Surgery, Huashan Hospital, Fudan University, Shanghai 200040, P.R. China

**Keywords:** carcinosarcoma, gallbladder, tumor thrombi

## Abstract

Gallbladder carcinosarcoma is one of the rarest subsets of gallbladder malignancies. The first case of carcinosarcoma of the gallbladder was reported in 1907. To date, <100 cases have been reported in the English literature. The present study reports a case of gallbladder carcinosarcoma accompanied with tumor thrombi, presenting as a soft tissue mass in the common bile duct and resulting in the obstruction and inflammation of the biliary tract. Initially, the patient was diagnosed with a gallbladder tumor and choledocholithiasis. No cases of carcinosarcoma of the gallbladder accompanied with bile duct tumor thrombus formation have been reported to date. A cholecystectomy with liver segmentectomy (S4a+S5) and a lymph node dissection were performed. The presence of a tumor thrombus in the common bile duct was confirmed by analysis of a frozen section during surgery. Resection of the extrahepatic bile duct and Roux-en-Y type hepatic cholangiojejunostomy were also performed. In addition, the gallbladder carcinosarcoma was observed to produce α-fetoprotein. The patient underwent an uneventful post-operative recovery and, to date, no clinical or radiological evidence of disease recurrence or metastasis has been identified. Carcinosarcoma of the gallbladder accompanied with tumor thrombi is extremely rare. Tumor thrombi in the common bile duct may easily be misdiagnosed as choledocholithiasis. The treatment and prognosis of gallbladder carcinosarcoma is similar to that of gallbladder carcinoma.

## Introduction

Carcinosarcoma is a rare tumor that is characterized by malignant epithelial and mesenchymal components. These tumors have been reported in a number of different organs, including the uterus, lung, esophagus, kidney and pancreas (1). Carcinosarcoma of the gallbladder is extremely rare. To date, <100 cases have been reported in the English literature. Here, we report a case of carcinosarcoma of the gallbladder accompanied with tumor thrombi. Written informed consent was obtained from the patient.

## Case report

### Clinical presentation and diagnosis of cancer

**Figure 1:**
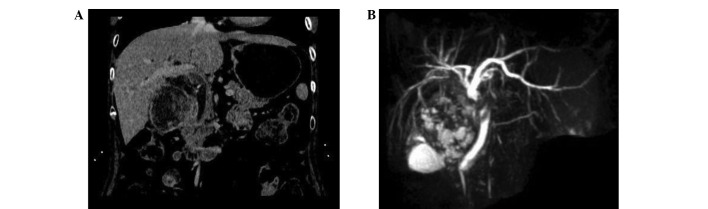
Pre-operative examination. Three-dimensional reconstruction by (A) CT scan and (B) magnetic resonance cholangiopancreatography revealed enlargement of the gallbladder with a large mass and tumor thrombi presenting as soft tissue mass in the common bile duct, with biliary dilatation above the obstruction. CT, computed tomography.

A 68-year-old female was admitted to the Huashan Hospital (Shanghai, China) with right upper abdominal pain and jaundice. The patient reported a past medical history of chronic cholecystitis, cholecystolithiasis and tuberculosis. A physical examination revealed a body temperature of 38.2°C and tenderness in the right upper quadrant of the abdomen. A laboratory analysis revealed leukocytosis (1.7×10^4^ cells/mm^3^). The tumor marker, serum carcinoembryonic antigen, carbohydrate antigen 19-9 and α-fetoprotein (AFP) levels were elevated to 33.67 *μ*g/l, 54.22 U/ml and 312.7 *μ*g/l, respectively. In addition, liver function tests revealed that the serum concentrations of alanine aminotransferase and alkaline phosphatase were increased to 411 U/l and 686 U/l, respectively. In addition, the total bilirubin levels were increased to 35.3 *μ*mol/l. Computed tomography (CT), three-dimensional reconstructions of this CT (Fig. 1A), ultrasonography and magnetic resonance cholangiopancreatography (Fig. 1B) indicated a large solid mass lesion in the gallbladder. Finally, a cholecystectomy with liver segmentectomy (S4a+S5) and a lymph node dissection were performed. The analysis of an intraoperative frozen section demonstrated that the soft tissue lump in the common bile duct was formed from tumor thrombi. Following this, a resection of the extrahepatic bile duct and a Roux-en-Y type hepatic cholangiojejunostomy were performed.

### Tumor characteristics

**Figure 2:**
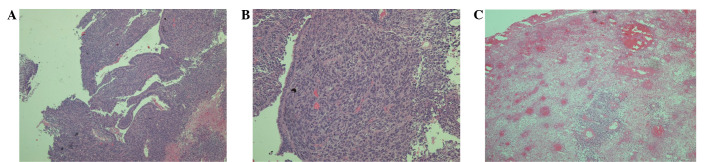
Neoplastic tissue of the gallbladder consisting of two patterns, a sarcomatous tissue and an adenocarcinoma. HE staining at magnifications (A) ×40, (B) ×200 and (C) ×100. Staining confirmed that the soft tissue mass was formed from tumor thrombi. HE, hematoxylin-eosin.

**Figure 3:**
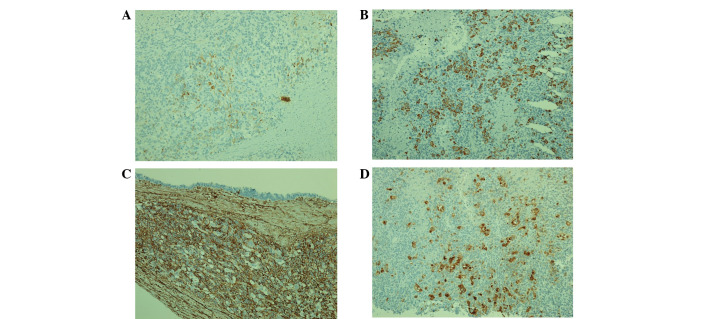
Immunohistochemical analysis of undifferentiated cells of the sarcomatous component in order to detect levels of (A) cytokeratin (anti-cytokeratin antibody staining) and (B) vimentin (anti-vimentin antibody staining), and an analysis of rhabdomyosarcomatous differentiation to detect levels of (C) desmin (anti-desmin antibody staining) and (D) myoglobin (anti-myoglobin antibody staining) (magnification, ×200).

Upon gross examination of the surgical specimen, a cross section revealed a 10×7×5-cm pedunculated polypoid solid mass with hemorrhagic and necrotic changes, protruding into the lumen of the gall-bladder. Histologically, the tumor was formed of two distinct components, namely poorly-differentiated tubular adenocarcinoma and sarcomatous tissue with rhabdomyosarcomatous differentiation (Fig. 2A and B). Following hematoxylin-eosin (HE) staining, the soft tissue mass from the common bile duct was diagnosed as a tumor thrombus (Fig. 2C). Immunohistochemical analysis revealed that the malignant epithelial component was positive for cytokeratin (Fig. 3). The spindle cells of the sarcomatous components, including the rhabdomyosarcomatous differentiation, were stained with antibodies against vimentin, desmin, myoglobin and cytokeratin (Fig. 3). The sample was negative for smooth muscle actin, CD56 and S-100. In addition, the carcinomatous and the sarcomatous areas were markedly positive for p53. The proliferation index, as detected by the Ki-67 labeling index (LI), was 70. The patient was discharged on post-operative day 11 and the serum AFP levels had decreased to normal levels after 1.5 months.

## Discussion

Carcinosarcomas, characterized by two intermingled epithelial and mesenchymal components within the same tissue, are an extremely atypical subset of gallbladder malignancies. The epithelial component usually consists of adenocarcinoma and, in rarer cases, elements of squamous cell, small cell and undifferentiated carcinomas are also observed. Although the sarcomatous component typically consists of undifferentiated spindle cells and heterologous elements, including osteosarcoma, chondrosarcoma, rhabdomyosarcoma and leiomyosarcoma, it may occasionally comprise part of the mesenchymal component (2–4). In the present case study, the carcinosarcoma of the gallbladder was composed of carcinomatous and sarcomatous portions and the sarcomatous differentiation revealed apparent rhabdomyosarcomatous differentiation.

The exact histogenesis of carcinosarcoma of the gall-bladder has not been clearly elucidated. At present, two opposing theories have been hypothesized to explain the origin of these morphologically diverse tumors. The multiclonal theory (convergence hypothesis) regards a carcinosarcoma as a collision tumor composed of the derivatives of two or more stem cells of separate epithelial and mesenchymal origin. The monoclonal theory (divergent hypothesis) proposes that carcinomatous and sarcomatous elements are derived from a single pluripotential stem cell that subsequently develops divergent differentiation along separate epithelial and mesenchymal pathways (5). Through the detection of associated gene fragments using molecular biology methods, specific studies have demonstrated that carcinosarcomas originating from the uterus, breast, lung and gastrointestinal tract are all monoclonal (6). Dacic *et al*(7) performed an extensive comparative genotypic analysis using microdissection to secure representative mesenchymal and epithelial components. The study found identical allelic losses shared by each tumor component, without discordant losses. This was consistent with the hypothesis that the carcinomatous and sarcomatous components of this neoplasm were derived from a single pluripotent stem cell and that the tumor was monoclonal. However, a larger series of cases and microdissection-based genotypic analyses of selected chromosomal loci must be performed to elucidate the precise mechanism of evolution of carcinosarcomas.

In the present case, the patient presented with acute biliary inflammation and obstructive jaundice as the initial complaint, as a result of an obstruction of the superior segment of the common bile duct caused by tumor thrombi. This presentation has rarely been reported in previous comparable studies (8). In the majority of these cases, the clinical symptoms included right upper abdominal pain and a mass, fever, jaundice, poor appetite, weight loss, general fatigue, nausea and vomiting. In addition, 75% of the cases presented with simultaneous cholecystolithiasis. The mechanism of tumor thrombi formation remains unclear and its clinical and pathological characteristics are undefined. We hypothesized that in the present study, a fragment of a necrotic tumor migrated to the common bile duct and caused an obstruction, or that a hemorrhage from the tumor may have partially or completely filled the biliary tract with tumor-containing blood clots. The only recognized treatment for gallbladder carcinosarcoma is surgery and there have been no reports of effective chemotherapy or radiotherapy for this tumor type. In the present study, the biliary tumor thrombi were successfully removed via choledochotomy. Following this, an extrahepatic bile duct resection and a reconstruction using a Roux-en-Y jejunal limb were performed.

AFP is a clinically useful and reliable marker for the diagnosis of primary hepatocellular carcinoma, hepatoblastoma and yolk-sac tumors (9). However, AFP-producing tumors arising from the gallbladder are extremely rare. In the present case study there was no evidence of liver metastasis and the serum AFP levels were elevated to 312.7 *μ*g/l; this decreased to within normal levels post-operatively. Hayashi *et al* hypothesized that AFP-producing carcinomas of the gallbladder more frequently metastasize to the liver, indicating a poorer prognosis than that of carcinomas that do not produce AFP (10). In addition, Ki-67, an indicator of proliferative activity, was previously reported to have a prognostic value. Kubota *et al* performed immunohistochemistry to investigate Ki-67 LI in 1 case of carcinosarcoma of the gallbladder and 11 cases of ordinary gallbladder adenocarcinoma classified as stage IV, to clarify the higher malignant proliferative potential of the former. The results revealed that the Ki-67 LI of the carcinosarcoma was 68, which was higher than the average level of ∼47 in the ordinary gallbladder adenocarcinomas (11).

In the current patient, the Ki-67 LI was 70, which was indicative of a higher proliferative and malignant potential and a subsequent poorer prognosis. However, the patient follow-up was uneventful and the individual remains healthy. A further detailed follow-up of this patient is vital in view of the aggressive biological behavior of this tumor.
